# Supplementary material for: A universal vector concept for a direct genotyping of transgenic organisms and a systematic creation of homozygous lines
Source: eLife. 2018 Mar 15;7:e31677. doi: 10.7554/eLife.31677 (PMC5854464; doi:10.7554/eLife.31677)
Supplement: Supplementary file 1. — F2 (mO-mC) founder females were mated with wild-type males and the progeny were scored. Segregation of 60% or fewer transgenic descendants was defined as the criterion for one insert. No deviators could be identified. [file elife-31677-supp1.docx]

| **Line** | **⚫⚫⚫** | **⚫⚫⚫** | **Total** |
| --- | --- | --- | --- |
| theoretical – one insertion | 50% | 50% | 100% |
| theoretical – two insertions^1^ | 25% | 75% | 100% |
| AGOC #1 | 54.7% (41) | 45.3% (34) | 75 |
| AGOC #2 | 46.2% (30) | 53.8% (35) | 65 |
| AGOC #3 | 61.9% (39) | 38.1% (24) | 63 |
| AGOC #4 | 55.8% (48) | 44.2% (38) | 86 |
| AGOC #5 | 55.1% (27) | 44.9% (22) | 49 |
| AGOC #6 | 56.3% (63) | 43.7% (49) | 112 |
| AGOC{ATub’#O(LA)-mEmerald} #1 | 47.1% (33) | 52.9% (37) | 70 |
| AGOC{Zen1’#O(LA)-mEmerald} #1 | 58.5% (38) | 41.5% (27) | 65 |
| AGOC{Zen1’#O(LA)-mEmerald} #2 | 46.6% (27) | 53.4% (31) | 58 |
| AGOC{Zen1’#O(LA)-mEmerald} #3 | 52.2% (35) | 47.8% (32) | 67 |
| AGOC{ARP5’#O(LA)-mEmerald} #1 | 54.3% (76) | 45.7% (64) | 140 |
| AGOC{ARP5’#O(LA)-mEmerald} #2 | 48.4% (75) | 51.6% (80) | 155 |
| AGOC{ATub’SiaTr-mEmerald} #1 | 51.9% (28) | 48.1% (26) | 54 |
| AGOC{ATub’SiaTr-mEmerald} #2 | 43.5% (30) | 56.5% (39) | 69 |
| AGOC{ATub’SiaTr-mEmerald} #3 | 48.5% (33) | 51.5% (35) | 68 |
| AGOC{ATub’H2B-mEmerald} #1 | 49.2% (31) | 50.8% (32) | 63 |
| AGOC{ATub’H2B-mEmerald} #2 | 64.4% (65) | 35.6% (36) | 101 |
| AGOC{ATub’H2B-mEmerald} #3 | 52.3% (46) | 47.7% (42) | 88 |
| AGOC{ATub’H2B-mEmerald} #4 | 55.2% (48) | 44.8% (39) | 87 |

^1^ this theoretical value corresponds to the case in which the insertions occur on different chromosomes, which is more likely than two independent inserts on the same chromosome.
