# Supplementary material for: A universal vector concept for a direct genotyping of transgenic organisms and a systematic creation of homozygous lines
Source: eLife. 2018 Mar 15;7:e31677. doi: 10.7554/eLife.31677 (PMC5854464; doi:10.7554/eLife.31677)
Supplement: Supplementary file 2. — Two F3 (mO-mC) pre-recombination hemizygous siblings were mated and the progeny were scored. Segregation of 70% or more transgenic descendants was defined as the criterion for homozygous viability. Deviators are marked bold. [file elife-31677-supp2.docx]

| **Line** | **⚫⚫⚫** | **⚫⚫⚫** | **Total** |
| --- | --- | --- | --- |
| theoretical – homozygous viable | 25.0% | 75.0% | 100% |
| theoretical – homozygous lethal | 33.3% | 66.7% | 100% |
| AGOC #1 | 23.7% (27) | 76.3% (87) | 114 |
| AGOC #2 | 29.5% (26) | 70.5% (62) | 88 |
| **AGOC #3** | **34.5% (20)** | **65.5% (38)** | **58** |
| AGOC #4 | 29.3% (36) | 70.7% (87) | 123 |
| AGOC #5 | 17.9% (19) | 82.1% (87) | 106 |
| AGOC #6 | 29.0% (18) | 71.0% (44) | 62 |
| **AGOC{ATub’#O(LA)-mEmerald} #1** | **33.8% (23)** | **66.2% (45)** | **68** |
| AGOC{Zen1’#O(LA)-mEmerald} #1 | 24.6% (17) | 75.4% (52) | 69 |
| AGOC{Zen1’#O(LA)-mEmerald} #2 | 25.0% (15) | 75.0% (45) | 60 |
| AGOC{Zen1’#O(LA)-mEmerald} #3 | 25.5% (12) | 74.5% (35) | 47 |
| AGOC{ARP5’#O(LA)-mEmerald} #1 | 21.7% (13) | 78.3% (47) | 60 |
| AGOC{ARP5’#O(LA)-mEmerald} #2 | 23.5% (12) | 76.5% (39) | 51 |
| AGOC{ATub’SiaTr-mEmerald} #1 | 28.9% (28) | 71.1% (69) | 97 |
| AGOC{ATub’SiaTr-mEmerald} #2 | 28.4% (19) | 71.6% (41) | 67 |
| AGOC{ATub’SiaTr-mEmerald} #3 | 24.6% (15) | 75.4% (46) | 61 |
| AGOC{ATub’H2B-mEmerald} #1 | 22.4% (19) | 77.6% (66) | 86 |
| AGOC{ATub’H2B-mEmerald} #2 | 23.4% (22) | 76.6% (72) | 94 |
| AGOC{ATub’H2B-mEmerald} #3 | 23.3% (27) | 76.7% (89) | 116 |
| **AGOC{ATub’H2B-mEmerald} #4** | **49.7% (73)** | **50.3% (72)** | **147** |
