# Supplementary material for: A universal vector concept for a direct genotyping of transgenic organisms and a systematic creation of homozygous lines
Source: eLife. 2018 Mar 15;7:e31677. doi: 10.7554/eLife.31677 (PMC5854464; doi:10.7554/eLife.31677)
Supplement: Supplementary file 3. — In the Junction column, the piggyBac TTAA insertion/excision target sequence is marked bold. [file elife-31677-supp3.docx]

| **Subline** | **Chromosome** | **Junction** | **Neighbors** | **Inverse primer pair and restriction**  **enzyme, control primer pair** |
| --- | --- | --- | --- | --- |
| AGOC #1 | Unknown  (reverse orientation) | 5’-AATAGTATTTTCGGTTGAAAAAGTAAGC**TTAA**  GCTGTTTTAGACCTAGGTTTTAGCATAG-3’ | *Cactus* (NW_001093503.1), approximately 28,000 bp in 5' with 2 gaps (head-to-head) | I-5’ with EcoRI, I1 |
| AGOC #2 | Not ChLGX or ChLGY | N.A. | N.A. | N.A. |
| AGOC #3 | ChLG8  (forward orientation) | 5'-ACATGTAAATGGGGGGCCCCATCATTCC**TTAA**  AGGTTGATTTATTTTGGGTGGGGGAGAA-3' | Intron 1 of *Sialin-like*, approximately 50 bp from Exon 1 (head-to-tail) | I-5’ with EcoRI, I2 |
| AGOC #4 | Not ChLGX or ChLGY | N.A. | N.A. | N.A. |
| AGOC #5 | ChLG10  (forward orientation) | 5'-AAAAAACCCTCTCTCCCACTGAACTCGT**TTAA**  TTGAATCAGCAGAGGAGAGAAAGACAGA-3' | Probable *Phenylalanine-tRNA ligase alpha subunit* (LOC655993), approximately 33,000 bp in 3' (tail-to-tail) | I-5’ with EcoRI, I3 |
| AGOC #6 | ChLG2  (reverse orientation) | 5'-GTCGGATCCAGCTGTTTGTCGTCGTACT**TTAA**  AGTGATCTGAATATTTTTCAAATAAACC-3' | Uncharacterized Loc (LOC659067), approximately 4,000 bp in 5' (tail-to-head) | I-5’ with BsrGI, I4 |
| AGOC{ATub'#O(LA)-mEmerald} #1 | ChLG2  (reverse orientation) | 5'-TCTAACGCCCACGATACACACACGATAA**TTAA**  AACGCTGTAAATAATGGAGAGGCTTTCG-3' | Probable *JmjC domain-containing histone demethylation protein 2C*, approximately 13,000 bp in 3' (tail-to-head) | I-3’ with SpeI, I5 |
| AGOC{Zen1'#O(LA)-mEmerald} #1 | ChLG3  (reverse orientation) | 5'-ATAGCAAATTATTATTTTAAGCTTTCCT**TTAA**  GTTGTGTTCGTAATTCTCGTTTGAAAGA-3' | *Activating transcription factor 7-interacting protein 1*, approximately 7,000 bp in 5' (head-to-head) | I-3’ with AatII, I6 |
| AGOC{Zen1'#O(LA)-mEmerald} #2 | ChLGX (allosomal) (reverse orientation) | 5'-TTTCCGACAATGGGTCCGTGACAATAGT**TTAA**  AAAGTCCGATCCTTCTCAGTGCGCCTAA-3' | *GTP-binding protein Rheb*, approximately 6,000 bp in 5' (tail-to-tail) | I-3’with EcoRI, I7 |
| AGOC{Zen1'#O(LA)-mEmerald} #3^1^ | Not ChLGX or ChLGY | N.A. | N.A. | N.A. |
| AGOC{ARP5'#O(LA)-mEmerald} #1 | ChLG9  (reverse orientation) | 5'-TGAACCCTTTGACATATTTTTTGTTAAA**TTAA**  ACTTGCCGTAATAATTGCCATAATTGCC-3'^2^ | *Membrane metallo-endopeptidase-like 1*, approximately 2,600 bp in 5' (tail-to-head) | I-5’ with EagI, control PCR not performed |
| AGOC{ARP5'#O(LA)-mEmerald} #2 | Not ChLGX or ChLGY | N.A. | N.A. | N.A. |
| AGOC{ATub'SiaTr-mEmerald} #1 | ChLG9  (reverse orientation) | 5'-GCTCCAACCACTGTTTCAGCTATTTAAT**TTAA**  AGCCCATAAATAACGATCGTTATCTCGT-3' | Intron 2 of *CUGBP ELAV-like family member 1*, approximately 3,500 bp from Exon 2 (head-to-tail) | I-5’ with SalI, I8 |
| AGOC{ATub'SiaTr-mEmerald} #2 | Not ChLGX or ChLGY | N.A. | N.A. | N.A. |
| AGOC{ATub'SiaTr-mEmerald} #3 | Not ChLGX or ChLGY | N.A. | N.A. | N.A. |
| AGOC{ATub'H2B-mEmerald} #1 | Unknown  (forward orientation) | 5'-GTCTCACATTTATCAGTCACTTGAAGTA**TTAA**  AGCTGCTAACATGTTAAAACCTTGACAA-3' | No genes within 100,000 bp | I-5’ with EcoRI, I9 |
| AGOC{ATub'H2B-mEmerald} #2 | Not ChLGX or ChLGY | N.A. | N.A. | N.A. |
| AGOC{ATub'H2B-mEmerald} #3 | Not ChLGX or ChLGY | N.A. | N.A. | N.A. |
| AGOC{ATub'H2B-mEmerald} #4 | Not ChLGX or ChLGY | N.A. | N.A. | N.A. |

^1^ the AGOC{Zen1'#O(LA)-mEmerald} #3 subline has a nested insertion, please see the Materials and methods section. ^2^ As the control polymerase chain reaction was not performed, the 28 bp upstream of the TTAA site derive from BeetleBase.
