# Supplementary material for: A universal vector concept for a direct genotyping of transgenic organisms and a systematic creation of homozygous lines
Source: eLife. 2018 Mar 15;7:e31677. doi: 10.7554/eLife.31677 (PMC5854464; doi:10.7554/eLife.31677)
Supplement: Supplementary file 4. — In this table, the F0 to F7 and their characteristics are summarized. [file elife-31677-supp4.docx]

| **Gen** | **Description / example terminology** | **Rationale** | **Cross** |
| --- | --- | --- | --- |
| F0 | wild-type pool  (PWAS strain) | The background line of choice, *i.e.* PWAS in our study. After a few weeks of adulthood, a replacement F0 is grown from a separate oviposition to keep the line alive. | *inter pares / en masse* |
| F1 | potential mosaics   - AGOC{ATub’#O(LA)-mEmerald #1 (mO-mC) mosaics | Created via injection of the respective transgenesis construct in conjunction with the ATub’piggyBac helper vector into preblastoderm embryos. Approximately 20% of the hatched larvae are F1 potential mosaics, but do not display any fluorescence and are identified by mating with wild types and screening the progeny. Approximately 1 to 2% of the F1 potential mosaics carry the transgene in the germline and give rise to several F2 (mO-mC) founders. | mating of all hatched larvae, *i.e.* potential mosaics with wild types |
| F2 | (mO-mC) founders   - AGOC{ATub’#O(LA)-mEmerald} #1 (mO-mC) founder | The first generation where all cells carry the transgene. For each F1 potential mosaic that produces transgenic progeny, one F2 (mO-mC) founder female is chosen to establish a subline, *i.e.* lines that carry the identical transgene at different genomic locations. Mating with wild-type males is necessary to (i) determine the number of inserts, (ii) establish a non-recombined F3 culture and (iii) get individuals of both genders for the homozygous viability cross. | to wild types |
| F3 | (mO-mC) pre-recombination hemizygotes   - AGOC{ATub’#O(LA)-mEmerald} #1 (mO-mC) | Up to this step, our scheme does not differ from most standard procedures to establish transgenic lines. Working cultures can be established from the F3 individuals that can be treated like classical transgenic lines and used in (preliminary) experiments. In parallel, systematic creation of homozygous lines can be initiated by mating F3 (mO-mC) pre-recombination hemizygous females with (mCe/mCe) homozygous helper males that express a nuclear-localized Cre recombinase. | to Cre recombinase-expressing helpers |
| F4 | (mCe; mO-mC) double hemizygotes (*i.e.* hybrids)   - AGOC{ATub’#O(LA)-mEmerald} #1 (mO-mC)   × ICE{HSP68’NLS-Cre} #1 (mCe) | The F4 (mCe; mO-mC) double hemizygotes are hybrids. Within this generation, recombination occurs and one of the two markers in cis configuration is excised. This process usually happens during later stages of development, so that the individuals typically display a patchy marker expression pattern in the compound eyes of adults (Supplementary Figure 3). | to wild types |
| F5 | (mO) and (mC) post-recombination hemizygotes   - AGOC{ATub’#O(LA)-mEmerald} #1 (mO) - AGOC{ATub’#O(LA)-mEmerald} #1 (mC) | In the F5 generation, the Cre-expressing transgene as well as one of the two markers are removed. As mO or mC are typically excised after most of the germline cells have differentiated, F5 (mO) and (mC) post-recombination hemizygotes are obtained from a single female-male pair. | mating of adult siblings that carry different markers |
| F6 | (mO/mC) heterozygotes   - AGOC{ATub’#O(LA)-mEmerald} #1 (mO/mC) | The F6 (mO/mC) heterozygotes can be identified as they carry both markers once again, but in trans configuration. This generation is homozygous for the non-marker part of the transgene. | mating of adult siblings that carry both markers |
| F7 | (mO/mO) and (mC/mC) homozygotes   - AGOC{ATub’#O(LA)-mEmerald} #1 (mO/mO) - AGOC{ATub’#O(LA)-mEmerald} #1 (mC/mC) | The F7 (mO/mO) and (mC/mC) homozygotes are selected by the omission of one marker. This is the final generation, from which continuous F7+ (mO/mO) and (mC/mC) homozygous cultures can be established. | *en masse* of adult siblings with the same marker to establish continuous F7+ homozygous cultures |
