# Supplementary material for: A universal vector concept for a direct genotyping of transgenic organisms and a systematic creation of homozygous lines
Source: eLife. 2018 Mar 15;7:e31677. doi: 10.7554/eLife.31677 (PMC5854464; doi:10.7554/eLife.31677)
Supplement: Supplementary file 5. — Bold entries mark progeny that were used in the subsequent cross. F6-S, F7-O and F7-C are control crosses. No significant differences between the arithmetic means and the theoretical Mendelian ratios were found. See Source Data 1 for raw scores ordered by transgenic sublines. [file elife-31677-supp5.docx]

| **Gen** | **Cross** | **Subline** | **Progeny** | | | | | | | | |
| --- | --- | --- | --- | --- | --- | --- | --- | --- | --- | --- | --- |
|  |  |  | ⚫⚫⚫ | ⚫⚫⚫ | ⚫⚫⚫ | ⚫⚫⚫ | ⚫⚫⚫ | ⚫⚫⚫ | ⚫⚫⚫ | ⚫⚫⚫ | **Total** |
| **F3** | 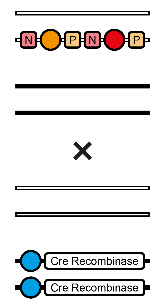 | Theoretical | - | 50.0% | - | - | - | - | - | **50.0%** | - |
|  |  | AGOC #5 sw. genders | - | 50.0% (35) | - | - | - | - | - | **50.0% (35)** | 70 |
|  |  | AGOC #6 sw. genders | - | 57.4% (62) | - | - | - | - | - | **42.6% (46)** | 108 |
|  |  | AGOC #5 alt. helper | - | 46.4% (26) | - | - | - | - | - | **53.6% (30)** | 56 |
|  |  | AGOC #6 alt. helper | - | 50.8% (65) | - | - | - | - | - | **49.2% (67)** | 132 |
|  |  | Arithmetic Mean | - | 51.1 ± 4.6% | - | - | - | - | - | **48.9 ± 4.6%** | 91.5 |
| **F4** | 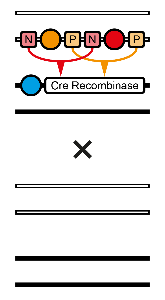 | Theoretical | 25.0% | 25.0% | **12.5%** | **12.5%** | 12.5% | 12.5% | - | - | - |
|  |  | AGOC #5 sw. genders | 14.4% (17) | 39.8% (47) | **10.2% (12)** | **24.6% (29)** | 4.2% (5) | 6.8% (8) | - | - | 118 |
|  |  | AGOC #6 sw. genders | 22.7% (30) | 35.6% (47) | **9.1% (12)** | **12.1% (16)** | 8.4% (11) | 12.1% (16) | - | - | 132 |
|  |  | AGOC #5 alt. helper | 7.5% (7) | 23.7% (22) | **9.7% (9)** | **14.0% (13)** | 20.4% (19) | 24.7% (23) | - | **-** | 93 |
|  |  | AGOC #6 alt. helper | 19.5% (18) | 47.8% (44) | **2.2% (2)** | **8.7% (8)** | 3.3% (3) | 18.5% (17) | - | - | 92 |
|  |  | Arithmetic Mean | 16.0 ± 6.6% | 36.7 ± 10.1% | **7.8 ± 3.8%** | **14.9 ± 6.7%** | 9.1 ± 7.9% | 15.5 ± 7.8% |  |  | 108.8 |
| **F5** | 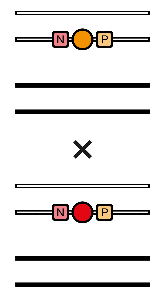 | Theoretical | 25.0% | - | 25.0% | 25.0% | - | - | **25.0%** | - | - |
|  |  | AGOC #5 sw. genders | 29.3% (29) | - | 31.3% (31) | 21.2% (21) | - | - | **18.2% (18)** | - | 99 |
|  |  | AGOC #6 sw. genders | 25.0% (26) | - | 26.9% (28) | 26.9% (28) | - | - | **21.2% (22)** | - | 104 |
|  |  | AGOC #5 alt. helper | 25.4% (17) | - | 20.9% (14) | 25.4% (17) | - | - | **28.3% (19)** | - | 67 |
|  |  | AGOC #6 alt. helper | 27.0% (24) | - | 23.6% (21) | 20.2% (18) | - | - | **29.2% (26)** | - | 89 |
|  |  | Arithmetic Mean | 26.7 ± 2.0% | - | 25.7 ± 4.5% | 23.4 ± 3.2% | - | - | **24.2 ± 5.4%** | - | 89.8 |
| **F6-S** | 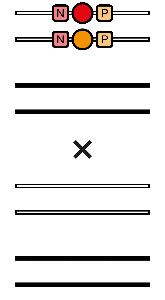 | Theoretical | - | - | 50.0% | 50.0% | - | - | - | - | - |
|  |  | AGOC #5 sw. genders | - | - | 47.9% (58) | 52.1% (63) | - | - | - | - | 121 |
|  |  | AGOC #6 sw. genders | - | - | 53.8% (28) | 46.2% (24) | - | - | - | - | 52 |
|  |  | AGOC #5 alt. helper | - | - | 55.7% (64) | 44.3% (51) | - | - | - | - | 115 |
|  |  | AGOC #6 alt. helper | - | - | 46.7% (28) | 53.3% (32) | - | - | - | - | 60 |
|  |  | Arithmetic Mean | - | - | 51.0 ± 4.4% | 49.0 ± 4.4% | - | - | - | - | 87.0 |
| **F6** | 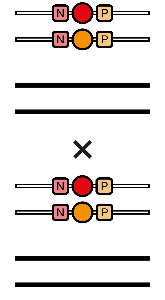 | Theoretical | - | - | **25.0%** | **25.0%** | - | - | 50.0% | - | - |
|  |  | AGOC #5 sw. genders | - | - | **25.4% (14)** | **18.2% (10)** | - | - | 56.4% (31) | - | 55 |
|  |  | AGOC #6 sw. genders | - | - | **21.2% (18)** | **27.0% (23)** | - | - | 51.8% (44) | - | 85 |
|  |  | AGOC #5 alt. helper | - | - | **31.1% (41)** | **23.5% (31)** | - | - | 45.4% (60) | - | 115 |
|  |  | AGOC #6 alt. helper | - | - | **40.6% (26)** | **18.8% (12)** | - | - | 40.6% (26) | - | 64 |
|  |  | Arithmetic Mean | - | - | **29.6 ± 8.4%** | **21.9 ± 4.2%** | - | - | 48.5 ± 7.0% | - | 79.8 |
| **F7-O** | 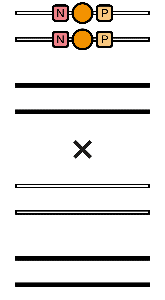 | Theoretical | - | - | 100% | - | - | - | - | - | - |
|  |  | AGOC #5 sw. genders | - | - | 100% (58) | - | - | - | - | - | 58 |
|  |  | AGOC #6 sw. genders | - | - | 100% (77) | - | - | - | - | - | 97 |
|  |  | AGOC #5 alt. helper | - | - | 100% (41) | - | - | - | - | - | 41 |
|  |  | AGOC #6 alt. helper | - | - | 100% (109) | - | - | - | - | - | 109 |
|  |  | Arithmetic Mean | - | - | 100 ± 0% | - | - | - | - | - | 76.3 |
| **F7-C** | 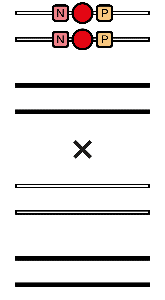 | Theoretical | - | - | - | 100% | - | - | - | - | - |
|  |  | AGOC #5 sw. genders | - | - | - | 100% (97) | - | - | - | - | 97 |
|  |  | AGOC #6 sw. genders | - | - | - | 100% (72) | - | - | - | - | 72 |
|  |  | AGOC #5 alt. helper | - | - | - | 100% (31) | - | - | - | - | 31 |
|  |  | AGOC #6 alt. helper | - | - | - | 100% (98) | - | - | - | - | 98) |
|  |  | Arithmetic Mean | - | - | - | 100 ± 0% | - | - | - | - | 74.5 |
