# Supplementary material for: A universal vector concept for a direct genotyping of transgenic organisms and a systematic creation of homozygous lines
Source: eLife. 2018 Mar 15;7:e31677. doi: 10.7554/eLife.31677 (PMC5854464; doi:10.7554/eLife.31677)
Supplement: Supplementary file 6. — Bold entries mark progeny that were used in the subsequent cross. F6-S, F7-O and F7-C are control crosses. No significant differences between the arithmetic means and the theoretical Mendelian ratios were found. See Source Data 1 for raw scores ordered by transgenic sublines. [file elife-31677-supp6.docx]

| **Gen** | **Cross** | **Subline** | **Progeny** | | | | | | | | |
| --- | --- | --- | --- | --- | --- | --- | --- | --- | --- | --- | --- |
|  |  |  | ⚫⚫⚫ | ⚫⚫⚫ | ⚫⚫⚫ | ⚫⚫⚫ | ⚫⚫⚫ | ⚫⚫⚫ | ⚫⚫⚫ | ⚫⚫⚫ | **Total** |
| F3 | 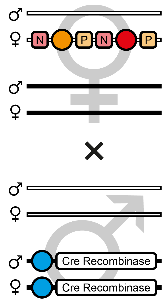 | Theoretical | - | 50.0% | - | - | - | - | - | **50.0%** | - |
|  |  | AGOC{ATub’#O(LA)-mEmerald} #1 | - | 44.8% (43) | - | - | - | - | - | **55.2% (53)** | 96 |
|  |  | AGOC{Zen1’#O(LA)-mEmerald} #1 | - | 57.4% (20) | - | - | - | - | - | **42.6% (27)** | 47 |
|  |  | AGOC{Zen1’#O(LA)-mEmerald} #2^1^ | - | 45.9% (45) | - | - | - | - | - | **54.1% (53)** | 98 |
|  |  | AGOC{Zen1’#O(LA)-mEmerald} #3 | - | 48.7% (55) | - | - | - | - | - | **51.3% (58)** | 113 |
|  |  | AGOC{ARP5’#O(LA)-mEmerald} #1 | - | 48.7% (37) | - | - | - | - | - | **51.3% (39)** | 76 |
|  |  | AGOC{ARP5’#O(LA)-mEmerald} #2 | - | 59.4% (38) | - | - | - | - | - | **40.6% (26)** | 64 |
|  |  | Arithmetic Mean | - | 50.8 ± 6.1% | - | - | - | - | - | **49.2 ± 6.1%** | 82.3 |
| F4 | 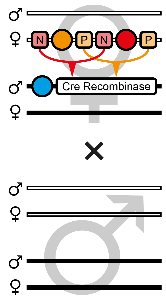 | Theoretical | 25.0% | 25.0% | **12.5%** | **12.5%** | 12.5% | 12.5% | - | - | - |
|  |  | AGOC{ATub’#O(LA)-mEmerald} #1 | 31.6% (38) | 29.2% (35) | **15.0% (18)** | **2.5% (3)** | 20.0% (18) | 1.7% (2) | - | - | 120 |
|  |  | AGOC{Zen1’#O(LA)-mEmerald} #1 | 32.4% (35) | 27.8% (30) | **6.5% (7)** | **14.8% (16)** | 7.4% (8) | 11.1% (12) | - | - | 108 |
|  |  | AGOC{Zen1’#O(LA)-mEmerald} #2^1^ | 19.6% (21) | 36.4% (39) | **5.6% (6)** | **15.0% (16)** | 5.6% (6) | 17.8% (19) | - | - | 107 |
|  |  | AGOC{Zen1’#O(LA)-mEmerald} #3 | 40.0% (38) | 24.2% (23) | **13.7% (13)** | **6.3% (6)** | 10.5% (10) | 5.3% (5) | - | - | 95 |
|  |  | AGOC{ARP5’#O(LA)-mEmerald} #1 | 19.6% (21) | 33.6% (36) | **10.3% (11)** | **9.4% (10)** | 12.1% (13) | 15.0% (16) | - | - | 107 |
|  |  | AGOC{ARP5’#O(LA)-mEmerald} #2 | 26.7% (27) | 28.7% (29) | **18.8% (19)** | **3.0% (3)** | 18.8% (19) | 4.0% (4) | - | - | 101 |
|  |  | Arithmetic Mean | 28.3 ± 8.0% | 30.0 ± 4.4% | **11.6 ± 5.1%** | **8.5 ± 5.6%** | 12.4 ± 5.9% | 9.2 ± 6.5% | - | - | 106.3 |
| F5 | 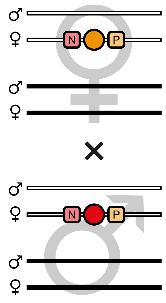 | Theoretical | 25.0% | - | 25.0% | 25.0% | - | - | **25.0%** | - | - |
|  |  | AGOC{ATub’#O(LA)-mEmerald} #1 | 27.2% (31) | - | 24.6% (28) | 26.3% (30) | - | - | **21.9% (25)** | - | 114 |
|  |  | AGOC{Zen1’#O(LA)-mEmerald} #1 | 26.6% (38) | - | 16.8% (24) | 33.5% (48) | - | - | **23.1% (33)** | - | 143 |
|  |  | AGOC{Zen1’#O(LA)-mEmerald} #2 | 20.2% (19) | - | 31.9% (30) | 22.4% (21) | - | - | **25.5% (24)** | - | 94 |
|  |  | AGOC{Zen1’#O(LA)-mEmerald} #3 | 22.7% (27) | - | 21.0% (25) | 34.5% (41) | - | - | **21.8% (26)** | - | 119 |
|  |  | AGOC{ARP5’#O(LA)-mEmerald} #1 | 25.8% (31) | - | 20.9% (25) | 28.3% (34) | - | - | **25.0% (30)** | - | 120 |
|  |  | AGOC{ARP5’#O(LA)-mEmerald} #2 | 27.1% (16) | - | 15.3% (9) | 28.8% (17) | - | - | **28.8% (17)** | - | 59 |
|  |  | Arithmetic Mean | 24.9 ± 2.9% | - | 21.8 ± 6.0% | 29.0 ± 4.5% | - | - | **24.3 ± 2.7%** | - | 108.2 |
| F6-S | 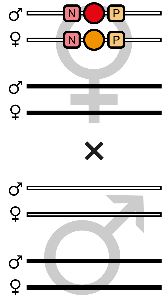 | Theoretical | - | - | 50.0% | 50.0% | - | - | - | - | - |
|  |  | AGOC{ATub’#O(LA)-mEmerald} #1 | - | - | 44.4% (36) | 55.6% (45) | - | - | - | - | 81 |
|  |  | AGOC{Zen1’#O(LA)-mEmerald} #1 | - | - | 43.9% (29) | 56.1% (37) | - | - | - | - | 66 |
|  |  | AGOC{Zen1’#O(LA)-mEmerald} #2^1^ | - | - | 48.8% (41) | 51.2% (53) | - | - | - | - | 84 |
|  |  | AGOC{Zen1’#O(LA)-mEmerald} #3 | - | - | 42.6% (55) | 57.4% (74) | - | - | - | - | 129 |
|  |  | AGOC{ARP5’#O(LA)-mEmerald} #1 | - | - | 55.6% (45) | 44.4% (36) | - | - | - | - | 81 |
|  |  | AGOC{ARP5’#O(LA)-mEmerald} #2 | - | - | 46..8% (37) | 53.2% (42) | - | - | - | - | 79 |
|  |  | Arithmetic Mean | - | - | 47.0 ± 4.8% | 53.0 ± 4.8% | - | - | - | - | 86.7 |
| F6 | 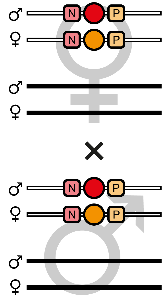 | Theoretical | - | - | **25.0%** | **25.0%** | - | - | 50.0% | - | - |
|  |  | AGOC{ATub’#O(LA)-mEmerald} #1 | - | - | **27.6% (27)** | **25.5% (25)** | - | - | 46.9% (46) | - | 98 |
|  |  | AGOC{Zen1’#O(LA)-mEmerald} #1 | - | - | **25.7% (37)** | **18.1% (26)** | - | - | 56.2% (81) | - | 144 |
|  |  | AGOC{Zen1’#O(LA)-mEmerald} #2^1^ | - | - | **42.2% (71)** | **39.3% (66)** | - | - | 18.6% (31) | - | 168 |
|  |  | AGOC{Zen1’#O(LA)-mEmerald} #3 | - | - | **26.8% (22)** | **26.8% (22)** | - | - | 46.4% (38) | - | 82 |
|  |  | AGOC{ARP5’#O(LA)-mEmerald} #1 | - | - | **14.0% (17)** | **30.3% (37)** | - | - | 55.7% (68) | - | 122 |
|  |  | AGOC{ARP5’#O(LA)-mEmerald} #2 | - | - | **23.6% (25)** | **24.5% (26)** | - | - | 51.9% (55) | - | 106 |
|  |  | Arithmetic Mean | - | - | **23.6 ± 5.5%** | **25.0 ± 4.5%** | - | - | 51.4 ± 4.7% | - | 110.4 |
| F7-O | 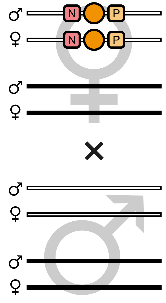 | Theoretical | - | - | 100% | - | - | - | - | - | - |
|  |  | AGOC{ATub’#O(LA)-mEmerald} #1 | - | - | 100% (99) | - | - | - | - | - | 99 |
|  |  | AGOC{Zen1’#O(LA)-mEmerald} #1 | - | - | 100% (89) | - | - | - | - | - | 89 |
|  |  | AGOC{Zen1’#O(LA)-mEmerald} #2^1^ | - | - | 100% (77) | - | - | - | - | - | 77 |
|  |  | AGOC{Zen1’#O(LA)-mEmerald} #3 | - | - | 100% (54) | - | - | - | - | - | 54 |
|  |  | AGOC{ARP5’#O(LA)-mEmerald} #1 | - | - | 100% (94) | - | - | - | - | - | 94 |
|  |  | AGOC{ARP5’#O(LA)-mEmerald} #2 | - | - | 100% (106) | - | - | - | - | - | 106 |
|  |  | Arithmetic Mean | - | - | 100 ± 0% | - | - | - | - | - | 86.5 |
| F7-C | 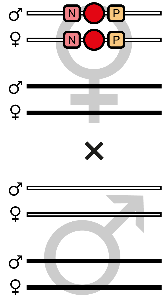 | Theoretical | - | - | - | 100% | - | - | - | - | - |
|  |  | AGOC{ATub’#O(LA)-mEmerald} #1 | - | - | - | 100% (64) | - | - | - | - | 64 |
|  |  | AGOC{Zen1’#O(LA)-mEmerald} #1 | - | - | - | 100% (101) | - | - | - | - | 101 |
|  |  | AGOC{Zen1’#O(LA)-mEmerald} #2^1^ | - | - | - | 100% (63) | - | - | - | - | 63 |
|  |  | AGOC{Zen1’#O(LA)-mEmerald} #3 | - | - | - | 100% (67) | - | - | - | - | 67 |
|  |  | AGOC{ARP5’#O(LA)-mEmerald} #1 | - | - | - | 100% (98) | - | - | - | - | 98 |
|  |  | AGOC{ARP5’#O(LA)-mEmerald} #2 | - | - | - | 100% (91) | - | - | - | - | 91 |
|  |  | Arithmetic Mean | - | - | - | 100 ± 0% | - | - | - | - | 80.7 |

^1^ the AGOC{Zen1’#O(LA)-mEmerald} #2 subline carries the transgene on the X allosome. Thus, two trans-generation crosses with two F6 (mO/mC) heterozygous females were performed, one to a F5 (mO) post-recombination hemizygous male and one to a F5 (mC) post-recombination hemizygous male. For both crosses, 84 individuals were scored (168 in total), the values shown are the sums of both crosses. See the Materials and methods section for details.
