# Supplementary material for: A universal vector concept for a direct genotyping of transgenic organisms and a systematic creation of homozygous lines
Source: eLife. 2018 Mar 15;7:e31677. doi: 10.7554/eLife.31677 (PMC5854464; doi:10.7554/eLife.31677)
Supplement: Supplementary file 7. — Bold entries mark progeny that were used in the subsequent cross. F6-S, F7-O and F7-C are control crosses. No significant differences between the arithmetic means and the theoretical Mendelian ratios were found. See Source Data 1 for raw scores ordered by transgenic sublines. [file elife-31677-supp7.docx]

| **Gen** | **Cross** | **Subline** | **Progeny** | | | | | | | | |
| --- | --- | --- | --- | --- | --- | --- | --- | --- | --- | --- | --- |
|  |  |  | ⚫⚫⚫ | ⚫⚫⚫ | ⚫⚫⚫ | ⚫⚫⚫ | ⚫⚫⚫ | ⚫⚫⚫ | ⚫⚫⚫ | ⚫⚫⚫ | **Total** |
| **F3** | 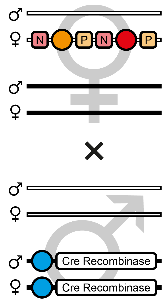 | Theoretical | - | 50.0% | - | - | - | - | - | **50.0%** | 100% |
|  |  | AGOC{ATub’SiaTr-mEmerald } #1 | - | 57.7% (41) | - | - | - | - | - | **42.3% (30)** | 71 |
|  |  | AGOC{ATub’SiaTr-mEmerald } #2 | - | 48.8% (39) | - | - | - | - | - | **51.2% (41)** | 80 |
|  |  | AGOC{ATub’SiaTr-mEmerald } #3 | - | 52.0% (39) | - | - | - | - | - | **48.0% (36)** | 75 |
|  |  | AGOC{ATub’H2B-mEmerald } #1 | - | 39.3% (22) | - | - | - | - | - | **60.7% (34)** | 56 |
|  |  | AGOC{ATub’H2B-mEmerald } #2 | - | 46.7% (28) | - | - | - | - | - | **53.3% (32)** | 60 |
|  |  | AGOC{ATub’H2B-mEmerald } #3 | - | 55.0% (55) | - | - | - | - | - | **45.0% (45)** | 100 |
|  |  | AGOC{ATub’H2B-mEmerald } #4 | - | 49.4% (44) | - | - | - | - | - | **50.6% (45)** | 89 |
|  |  | Arithmetic Mean | - | 49.8 ± 6.0% | - | - | - | - | - | **50.2 ± 6.0%** | 77.3 |
| **F4** | 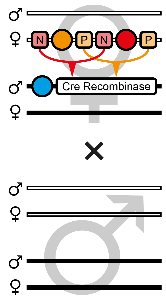 | Theoretical | 25.0% | 25.0% | **12.5%** | **12.5%** | 12.5% | 12.5% | - | - | - |
|  |  | AGOC{ATub’SiaTr-mEmerald } #1 | 29.8% (31) | 20.2% (21) | **23.1% (24)** | **2.9% (3)** | 21.1% (22) | 2.9% (3) | - | - | 104 |
|  |  | AGOC{ATub’SiaTr-mEmerald } #2 | 24.8% (28) | 30.1% (34) | **4.4% (5)** | **19.4% (22)** | 7.1% (8) | 14.2% (16) | - | - | 113 |
|  |  | AGOC{ATub’SiaTr-mEmerald } #3 | 37.9% (36) | 25.2% (24) | **5.3% (5)** | **9.5% (9)** | 15.8% (15) | 6.3% (6) | - | - | 95 |
|  |  | AGOC{ATub’H2B-mEmerald } #1 | 29.4% (32) | 23.9% (26) | **11.9% (13)** | **16.5% (18)** | 5.5% (6) | 12.8% (14) | - | - | 109 |
|  |  | AGOC{ATub’H2B-mEmerald } #2 | 21.8% (24) | 36.4% (40) | **22.7% (25)** | **10.0% (11)** | 3.6% (4) | 5.5% (6) | - | - | 110 |
|  |  | AGOC{ATub’H2B-mEmerald } #3 | 35.8% (34) | 25.3% (24) | **10.5% (10)** | **8.4% (8)** | 14.7% (14) | 5.3% (5) | - | - | 95 |
|  |  | AGOC{ATub’H2B-mEmerald } #4 | 21.8% (24) | 31.8% (35) | **21.8% (24)** | **13.6% (15)** | 6.4% (7) | 4.6% (5) | - | - | 110 |
|  |  | Arithmetic Mean | 28.7 ± 6,4% | 27.6 ± 5.5% | **14.2 ± 8.2%** | **11.5 ± 5.5%** | 10.6 ± 6.6% | 7.4 ± 4.3% | - | - | 105.1 |
| **F5** | 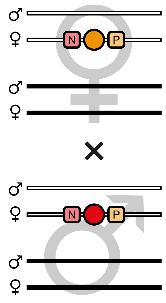 | Theoretical | 25.0% | - | 25.0% | 25.0% | - | - | **25.0%** | - | - |
|  |  | AGOC{ATub’SiaTr-mEmerald } #1 | 21.6% (21) | - | 26.8% (26) | 28.9% (28) | - | - | **22.7% (22)** | - | 97 |
|  |  | AGOC{ATub’SiaTr-mEmerald } #2 | 20.7/% (24) | - | 30.2% (35) | 26.7% (31) | - | - | **22.4% (26)** | - | 116 |
|  |  | AGOC{ATub’SiaTr-mEmerald } #3 | 19.3% (16) | - | 30.1% (25) | 28.9% (24) | - | - | **21.7% (18)** | - | 83 |
|  |  | AGOC{ATub’H2B-mEmerald } #1 | 29.2% (33) | - | 28.3% (32) | 23.9% (27) | - | - | **18.6% (21)** | - | 113 |
|  |  | AGOC{ATub’H2B-mEmerald } #2 | 28.6% (30) | - | 19.0% (20) | 21.0% (22) | - | - | **31.4% (33)** | - | 105 |
|  |  | AGOC{ATub’H2B-mEmerald } #3 | 22.6% (21) | - | 25.8% (24) | 20.4% (19) | - | - | **31.2% (29)** | - | 93 |
|  |  | AGOC{ATub’H2B-mEmerald } #4 | 26.8% (19) | - | 21.1% (15) | 26.8% (19) | - | - | **25.3% (18)** | - | 71 |
|  |  | Arithmetic Mean | 24.1 ± 4.0% | - | 25.9 ± 4.4% | 25.2 ± 3.5% | - | - | **24.8 ± 4.9%** | - | 96.9 |
| **F6-S** | 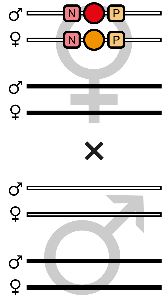 | Theoretical | - | - | 50.0% | 50.0% | - | - | - | - | - |
|  |  | AGOC{ATub’SiaTr-mEmerald } #1 | - | - | 48.2% (52) | 51.8% (56) | - | - | - | - | 108 |
|  |  | AGOC{ATub’SiaTr-mEmerald } #2 | - | - | 52.8% (28) | 47.2% (25) | - | - | - | - | 53 |
|  |  | AGOC{ATub’SiaTr-mEmerald } #3 | - | - | 44.4% (44) | 55.6% (55) | - | - | - | - | 99 |
|  |  | AGOC{ATub’H2B-mEmerald } #1 | - | - | 40.6% (28) | 59.4% (41) | - | - | - | - | 69 |
|  |  | AGOC{ATub’H2B-mEmerald } #2 | - | - | 48.6% (35) | 51.4% (37) | - | - | - | - | 72 |
|  |  | AGOC{ATub’H2B-mEmerald } #3 | - | - | 50.9% (55) | 49.1% (53) | - | - | - | - | 108 |
|  |  | AGOC{ATub’H2B-mEmerald } #4^1^ | - | - | - | - | - | - | - | - | - |
|  |  | Arithmetic Mean | - | - | 47.6 ± 4.4% | 52.4 ± 4.4% | - | - | - | - | 84.8 |
| **F6** | 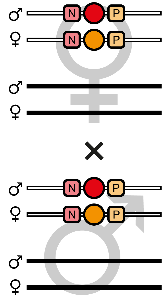 | Theoretical | - | - | **25.0%** | **25.0%** | - | - | 50.0% | - | - |
|  |  | AGOC{ATub’SiaTr-mEmerald } #1 | - | - | **27.9% (29)** | **27.9% (29)** | - | - | 44.2% (46) | - | 104 |
|  |  | AGOC{ATub’SiaTr-mEmerald } #2 | - | - | **28.0% (42)** | **27.3% (41)** | - | - | 44.7% (67) | - | 150 |
|  |  | AGOC{ATub’SiaTr-mEmerald } #3 | - | - | **20.8% (10)** | **18.8% (9)** | - | - | 60.4% (29) | - | 48 |
|  |  | AGOC{ATub’H2B-mEmerald } #1 | - | - | **41.1% (23)** | **12.5% (7)** | - | - | 46.4% (26) | - | 56 |
|  |  | AGOC{ATub’H2B-mEmerald } #2 | - | - | **25.5% (27)** | **26.4% (28)** | - | - | 48.1% (51) | - | 106 |
|  |  | AGOC{ATub’H2B-mEmerald } #3 | - | - | **28.3% (39)** | **27.5% (38)** | - | - | 44.2% (61) | - | 138 |
|  |  | AGOC{ATub’H2B-mEmerald } #4^1^ | - | - | **-** | **-** | - | - | - | - | - |
|  |  | Arithmetic Mean | - | - | **28.6 ± 6.8%** | **23.4 ± 6.3%** | - | - | 48.0 ± 6.3% | - | 100.3 |
| **F7-O** | 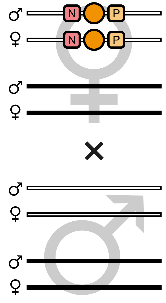 | Theoretical | - | - | 100% | - | - | - | - | - | - |
|  |  | AGOC{ATub’SiaTr-mEmerald } #1 | - | - | 100% (57) | - | - | - | - | - | 57 |
|  |  | AGOC{ATub’SiaTr-mEmerald } #2 | - | - | 100% (51) | - | - | - | - | - | 51 |
|  |  | AGOC{ATub’SiaTr-mEmerald } #3 | - | - | 100% (59) | - | - | - | - | - | 59 |
|  |  | AGOC{ATub’H2B-mEmerald } #1 | - | - | 100% (103) | - | - | - | - | - | 103 |
|  |  | AGOC{ATub’H2B-mEmerald } #2 | - | - | 100% (155) | - | - | - | - | - | 155 |
|  |  | AGOC{ATub’H2B-mEmerald } #3 | - | - | 100% (103) | - | - | - | - | - | 103 |
|  |  | AGOC{ATub’H2B-mEmerald } #4^1^ | - | - | - | - | - | - | - | - | - |
|  |  | Arithmetic Mean | - | - | 100 ± 0% | - | - | - | - | - | 88.0 |
| **F7-C** | 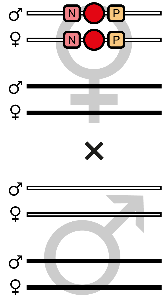 | Theoretical | - | - | - | 100% | - | - | - | - | - |
|  |  | AGOC{ATub’SiaTr-mEmerald } #1 | - | - | - | 100% (48) | - | - | - | - | 48 |
|  |  | AGOC{ATub’SiaTr-mEmerald } #2 | - | - | - | 100% (66) | - | - | - | - | 66 |
|  |  | AGOC{ATub’SiaTr-mEmerald } #3 | - | - | - | 100% (73) | - | - | - | - | 73 |
|  |  | AGOC{ATub’H2B-mEmerald } #1 | - | - | - | 100% (134) | - | - | - | - | 134 |
|  |  | AGOC{ATub’H2B-mEmerald } #2 | - | - | - | 100% (86) | - | - | - | - | 86 |
|  |  | AGOC{ATub’H2B-mEmerald } #3 | - | - | - | 100% (71) | - | - | - | - | 71 |
|  |  | AGOC{ATub’H2B-mEmerald } #4^1^ | - | - | - | **-** | - | - | - | - | - |
|  |  | Arithmetic Mean | - | - | - | 100 ± 0% | - | - | - | - | 79.7 |

^1^ the AGOC{ATub’H2B-mEmerald} #4 subline did not produce any progeny when F6 (mO/mC) heterozygotes were mated (n = 12), when F6 (mO/mC) heterozygous females and wild-type males were mated (n = 12), and, as an additional control, when F6 (mO/mC) heterozygous males and wild-type females were mated (n = 8). It was therefore assumed that this subline is sterile when both chromosomes carry the transgene. In the F6-S, F6, F7-O and F7-C crosses, it was left out of the mean calculations.
