# Supplementary material for: A universal vector concept for a direct genotyping of transgenic organisms and a systematic creation of homozygous lines
Source: eLife. 2018 Mar 15;7:e31677. doi: 10.7554/eLife.31677 (PMC5854464; doi:10.7554/eLife.31677)
Supplement: Supplementary file 8. — The 24 vectors used/created in this study listed in order of their type. Numbers in square brackets within the Source/molecular cloning column refer to the respective entry. See also (Figure 1—figure supplement 4). [file elife-31677-supp8.docx]

| **#** | **Vector** | **Type** | **Size** | **Res** | **Source / molecular cloning** | **Availability** |
| --- | --- | --- | --- | --- | --- | --- |
| [01] | pUC57[AGOC] | gene synthesis (Genewiz) | 5,678 bp | KanR | Ordered from Genewiz (3,277 bp *de novo* synthetized sequence inserted into the pUC57-Kan backbone via NdeI / PstI | our laboratory |
| [02] | pGS[#P’#O(LA)-mEmerald] | gene synthesis (Invitrogen) | 4,111 bp | KanR | Ordered from Invitrogen (1,833 bp *de novo* synthetized sequence inserted into the pMK-RQ backbone via SfiI | our laboratory |
| [03] | pGS[ACOS] | gene synthesis (Invitrogen) | 4,928 bp | KanR | Ordered from Invitrogen (2,650 bp *de novo* synthetized sequence inserted into the pMK-RQ backbone via SfiI | our laboratory |
| [04] | pBSII-IFP-ORF | previously published | 4,971 bp | AmpR | Kind gift from Malcom Fraser, vector described in ^67^ | Malcom Fraser |
| [05] | pTriEx-HTNC | previously published | 6,757 bp | AmpR | Ordered from Addgene (#13763), vector described in ^12^ | Addgene (#13763) |
| [06] | pTC-ATub’-GEM-T Easy | promoter library vector | 3,633 bp | AmpR | ATub’ amplified from genomic DNA with primer pair C3, A-tailed and inserted into pGEM-T Easy (copy & paste) | our laboratory |
| [07] | pTC-HSP68’-GEM-T Easy | promoter library vector | 3,778 bp | AmpR | HSP68’ amplified from genomic DNA with primer pair C4, A-tailed and inserted into pGEM-T Easy (copy & paste) | our laboratory |
| [08] | pTC-Zen1’-GEM-T Easy | promoter library vector | 4,599 bp | AmpR | Zen1’ amplified from genomic DNA with primer pair C5, A-tailed and inserted into pGEM-T Easy (copy & paste) | our laboratory |
| [09] | pTC-ARP5’-GEM-T Easy | promoter library vector | 5,516 bp | AmpR | ARP5’ amplified from genomic DNA with primer pair C6, A-tailed and inserted into pGEM-T Easy (copy & paste) | our laboratory |
| [10] | pTC-’SiaTr-GEM-T Easy | ORF library vector | 4,358 bp | AmpR | ’SiaTr amplified from cDNA with primer pair C7, A-tailed and inserted into pGEM-T Easy (copy & paste) | our laboratory |
| [11] | pTC-’H2B-GEM-T Easy | ORF library vector | 3,389 bp | AmpR | ’H2B amplified from cDNA with primer pair C8, A-tailed and inserted into pGEM-T Easy (copy & paste) | our laboratory |
| [12] | pTC-HSP68’NLS-Cre-GEM-T Easy | promoter / ORF library vector | 5,073 bp | AmpR | HSP68‘ and ’NLS-Cre amplified from [05] and [07], joined via fusion PCR with primer pair C9, A-tailed and inserted into pGEM-T Easy (fusion PCR) | our laboratory |
| [13] | pTC-ATub’H2B-GEM-T Easy | promoter / ORF library vector | 4,010 bp | AmpR | ATub’ and ’H2B amplified from [06] and [11], joined via fusion PCR with primer pair C10, A-tailed and inserted into pGEM-T Easy (fusion PCR) | our laboratory |
| [14] | pATub’piggyBac | helper | 5,511 bp | AmpR | ATub’ and a fragment of ’piggyBac fusion PCR with primer pair C2 and inserted into the unique SalI / BglII sites | Addgene |
| [15] | pICE{HSP68’NLS-Cre} | helper / transformation | 5,709 bp | KanR | 3×P3’mCerulean2 cassette amplified from [03] with primer pair C11 and inserted into the AflII / AvrII sites of [16] | Addgene |
| [16] | pAVOIAF{#1–#2–HSP68’NLS-Cre–mC} | intermediate | 5,781 bp | KanR | HSP68’NLS-Cre cassette cut from [12] with NheI / XhoI and inserted into the respective sites of [19] | our laboratory |
| [17] | pAGOC{#P’#O(LA)-mEmerald} | intermediate | 6,852 bp | KanR | #P’#O(LA)-mEmerald cassette cut from [02] with HindIII / XbaI and inserted into the respective sites of [19] | Addgene |
| [18] | pAGOC{#P’SiaTr-mEmerald} | intermediate | 8,106 bp | KanR | ’SiaTr amplified from [10] with primer pair C12 and inserted into the FseI / NotI sites of [17] | our laboratory |
| [19] | pAGOC | intermediate / transformation | 5,049 bp | KanR | AGOC cassette amplified from [01] with primer pair C1 and inserted into upstream (AatII) and downstream (PciI) sites of the same vector for size reduction | Addgene |
| [20] | pAGOC{ATub’#O(LA)-mEmerald} | transformation | 7,400 bp | KanR | ATub’ amplified from [06] with primer pair C13, digested with AscI / BsmBI and inserted into the respective sites of [17], which was digested with BtgZI | our laboratory |
| [21] | pAGOC{ATub’SiaTr(LA)-mEmerald} | transformation | 8,654 bp | KanR | ATub’ amplified from 06 with primer pair C13, digested with AscI / BsmBI and inserted into the respective sites of [18], which was digested with BtgZI | our laboratory |
| [22] | pAGOC{ATub’H2B-mEmerald} | transformation | 7,718 bp | KanR | ATub’H2B cassette cut from [13] with AscI / NotI and inserted into the respective sites of [17] | our laboratory |
| [23] | pAGOC{Zen1’#O(LA)-mEmerald} | transformation | 8.367 bp | KanR | Zen1’ amplified from [06] with primer pair C14, digested with AscI / BsaI and inserted into the respective sites of [17], which was digested with BtgZI | our laboratory |
| [24] | pAGOC{ARP5’#O(LA)-mEmerald} | transformation | 9,276 bp | KanR | ARP5’ amplified from 06 with primer pair C15, digested with AscI / BsaI and inserted into the respective sites of [17], which was digested with BtgZI | our laboratory |
| [25] | pAVOIAF{#1–#2–#3–#4} | hypothetical vector | 2,485 bp | KanR | Vector was designed *in silico* based on the pUC57 backbone. The four-slot cloning site is located between the AatII and PciI sites | - |
