# Supplementary material for: A universal vector concept for a direct genotyping of transgenic organisms and a systematic creation of homozygous lines
Source: eLife. 2018 Mar 15;7:e31677. doi: 10.7554/eLife.31677 (PMC5854464; doi:10.7554/eLife.31677)
Supplement: Supplementary file 9. — Primer pairs are listed in order of appearance in the Materials and methods section and Supplementary file 3. The Applied Biosciences web calculator (www6.appliedbiosystems.com/support/techtools/calc) was used to calculate the melting temperature TM. In case of primers with overhangs, the TM was only calculated for the annealing part. Primer that introduce a restriction enzyme site also carry a 6 bp (5’-AAATTT-3’) buffer at the 5’ end. Several primers have been used in multiple inverse PCRs and are therefore also listed multiple times, as annotated within the Comment column. ExPCR, extraction polymerase chain reaction; SiRePCR, size reduction polymerase chain reaction; FuPCR, fusion polymerase chain reaction; TrPCR, transfer polymerase chain reaction; InvPCR, inverse polymerase chain reaction; ConPCR, control polymerase chain reaction; FD, forward; RV, reverse. [file elife-31677-supp9.docx]

| **Primer pair** | **#** | **Primer name** | **Primer sequence** | **Comment** | **Length** | **T_M_** |
| --- | --- | --- | --- | --- | --- | --- |
| C1 | C1-1 | TC ATub’ ExPCR FD | 5’-AGATGTCTATGTATCTCCCGTGAAAC-3’ | - | 26 bp | 59.7°C |
|  | C1-2 | TC ATub’ ExPCR RV | 5’-GAAATGGGATAACTTACCATTTTGG-3’ | - | 25 bp | 59.7°C |
| C2 | C2-1 | TC Zen1’ ExPCR FD | 5’-AAATGCATGATTAACTGATCATGTGAC-3’ | - | 27 bp | 61.1°C |
|  | C2-2 | TC Zen1’ ExPCR RV | 5’-CAAATTGGGAGTAAGACATTTCGTC-3’ | - | 25 bp | 60.8°C |
| C3 | C3-1 | TC ARP5’ ExPCR FD | 5’-CAACGATTAAATCTCCGTGATAACAC-3’ | - | 26 bp | 60.7°C |
|  | C3-2 | TC ARP5’ ExPCR RV | 5’-CAAGTCTTTGAATTCGAGAATTTCC-3’ | - | 25 bp | 60.3°C |
| C4 | C4-1 | TC HSP68’ ExPCR FD | 5’-GAAAGTCGGGACCGAAGGTC-3’ | - | 20 bp | 61.7°C |
|  | C4-2 | TC HSP68’ ExPCR RV | 5’-CAATACCAACTGCTGGAGATTTCAC-3’ | - | 25 bp | 61.8°C |
| C5 | C5-1 | TC ’SiaTr ExPCR FD | 5’-ATGAGAGCTCTAGTGGTATCCATATGG-3’ | NCBI Reference Sequence: XM_963657.3 | 27 bp | 60.8°C |
|  | C5-2 | TC ’SiaTr ExPCR RV | 5’-CGTCTAAAAATTCACTCCAAAATTTCTC-3’ | NCBI Reference Sequence: XM_963657.3 | 28 bp | 61.4°C |
| C6 | C6-1 | TC ’H2B ExPCR FD | 5’-ATGCCACCAAAGACAAGC-3’ | NCBI Reference Sequences: XM_008203251.2 | 18 bp | 55.5°C |
|  | C6-2 | TC ’H2B ExPCR RV | 5’-CTATTTTGAACTTGTGTATTTGGTG-3’ | NCBI Reference Sequences: XM_008203251.2 | 25 bp | 55.7°C |
| C7 | C7-1 | HSP68’ and ’NLS-Cre FuPCR FD | 5’-AAATTT**GCTAGC**AAAGTCGGGACCGAAGGTCTTAATAAAAA-3’ | overhang underlined, NheI site bold | 31 bp | 66.0°C |
|  | C7-2 | HSP68’ and ’NLS-Cre FuPCR RV | 5’-CACCTTCCTCTTCTTCTTGGGGGC**CAT**TTTTGCACTTTTGGATTTACTTTG-3’ | ‘NLS-Cre overhang underlined, start codon bold | 51 bp | 61.8°C |
|  | C7-3 | ’NLS-Cre and HSP68’ FuPCR FD | 5’-AAAGTAAATCCAAAAGTGCAAAA**ATG**GCCCCCAAGAAGAAGAGGAAGGTGTCCAATTTAC-3’ | HSP68’ overhang underlined, start codon bold | 61 bp | 76.6°C |
|  | C7-4 | ’NLS-Cre and HSP68’ FuPCR RV | 5’-AAATTT**CTCGAG**TAAGATACATTGATGAGTTTGGACAAACCACAACTAG-3’ | overhang underlined, XhoI site bold | 40 bp | 68.2°C |
| C8 | C8-1 | ATub’ and ’H2B FuPCR FD | 5’-AAATTT**GGCGCGCC**AGATGTCTATGTATCTCCCGTGAAAC-3’ | overhang underlined, AscI site bold | 26 bp | 59.7°C |
|  | C8-2 | ATub’ and ’H2B FuPCR RV | 5’-CGCTTGTCTTTGGTGG**CAT**TTTGGTAGTTGAGTTTTACAAATTAC-3’ | ‘H2B overhang underlined, start codon bold | 45 bp | 57.8°C |
|  | C8-3 | ’H2B and ATub’ FuPCR FD | 5’-GTAAAACTCAACTACCAAA**ATG**CCACCAAAGACAAGCGGTAAAG-3’ | ATub’ overhang underlined, start codon bold | 44 bp | 66.8°C |
|  | C8-4 | ’H2B and ATub’ FuPCR RV | 5’-AAATTT**GCGGCCGC**TTTTGAACTTGTGTATTTGGTGACG-3’ | overhang underlined, NotI site bold | 25 bp | 60.9°C |
| C9 | C9-1 | AGOC SiRePCR FD | 5’-AAATTT**GACGTC**TTAACCCTAGAAAGATAATCATATTGTGACGTAC-3’ | overhang underlined, AatII site bold | 34 bp | 62.6°C |
|  | C9-2 | AGOC SiRePCR RV | 5’-AAATTT**ACATGT**TTAACCCTAGAAAGATAGTCTGCGTAAAATTG-3’ | overhang underlined, PciI site bold | 32 bp | 63.6°C |
| C10 | C10-1 | ATub’ TrPCR FD | 5’-AAATTT**GGCGCGCC**AGATGTCTATGTATCTCCCGTGAAAC-3’ | overhang underlined, AscI site bold | 40 bp | 59.7°C |
|  | C10-2 | ATub’ TrPCR FD | 5’-AAATTT**CGTCTC**ACCATTTTGGTAGTTGAGTTTTACAAATTAC-3’ | overhang underlined, BsmBI site bold | 43 bp | 60.4°C |
| C11 | C11-1 | Zen1’ TrPCR FD | 5’-AAATTT**GGCGCGCC**AAATGCATGATTAACTGATCATGTGAC-3’ | overhang underlined, AscI site bold | 41 bp | 61.1°C |
|  | C11-2 | Zen1’ TrPCR RV | 5’-AAATTT**GGTCTC**ACCATTTCGTCAAAGTGAAGTTTGC-3’ | overhang underlined, BsaI site bold | 37 bp | 59.6°C |
| C12 | C12-1 | ARP5’ TrPCR FD | 5’-AAATTT**GGCGCGCC**CAACGATTAAATCTCCGTGATAACAC-3’ | overhang underlined, AscI site bold | 40 bp | 60.7°C |
|  | C12-2 | ARP5’ TrPCR RV | 5’-AAATTT**GGTCTC**ACCATTTTTTGTGGGTTAAGTGAC-3’ | overhang underlined, BsaI site bold | 40 bp | 54.9°C |
| C13 | C13-1 | ’SiaTr TrPCR FD | 5’-AAATTT**GGCCGGCC**AAAATGAGAGCTCTAGTGGTATCCATATGG-3’ | overhang underlined, FseI site bold | 44 bp | 60.8°C |
|  | C13-2 | ’SiaTr TrPCR RV | 5’-AAATTT**GCGGCCGC**ACATTTAAAATTTTTGAAACCCAAGATTC-3’ | overhang underlined, NotI site bold | 43 bp | 61.6°C |
| C14 | C14-1 | 3xP3’mCerulean TrPCR FD | 5’-AAATTT**CTTAAG**GTTCCCACAATGGTTAATTCGAG-3’ | overhang underlined, AflII site bold | 35 bp | 60.4°C |
|  | C14-2 | 3xP3’mCerulean TrPCR RV | 5’-AAATTT**CCTAGG**TAAGATACATTGATGAGTTTGG-3’ | overhang underlined, AvrII site bold | 34 bp | 50.7°C |
| C15 | C15-1 | ATub’ and ’piggyBac FuPCR FD | 5’-AAATTT**GTCGAC**AGATGTCTATGTATCTCCCGTGAAAC-3’ | overhang underlined, SalI site bold | 38 bp | 59.7°C |
|  | C15-2 | ATub’ and ’piggyBac FuPCR RV | 5’-CTCATCGTCTAAAGAACTACC**CAT**TTTGGTAGTTGAGTTTTACAAATTAC-3’ | piggyBac ORF overhang underlined, start codon bold | 50 bp | 57.8°C |
|  | C15-3 | ’piggyBac and ATub’ FuPCR FD | 5’-GTAAAACTCAACTACCAAA**ATG**GGTAGTTCTTTAGACGATGAGC-3’ | ATub’ overhang underlined, start codon bold | 44 bp | 59.9°C |
|  | C15-4 | ’piggyBac and ATub’ FuPCR RV | 5’-AAATTT**AGATCT**AGATCTGACAATGTTCAGTGCAGAG-3’ | overhang underlined, BglII site bold | 25 bp | 60.7°C |
| I-3’ | I-3’-1 | AGOC 3’ TR InvPCR FD | 5’-AAAACTTGTTTAAACCCCGGG-3’ | - | 21 bp | 59.9°C |
|  | I-3’-2 | AGOC 3’ TR InvPCR RV | 5’-GCATGATTATCTTTAACGTACGTCAC-3’ | - | 26 bp | 58.9°C |
| I-5’ | I-5’-1 | AGOC 5’ TR InvPCR FD | 5’-CGCTATTTAGAAAGAGAGAGCAATATTTC-3’ | - | 29 bp | 60.4°C |
|  | I-5’-2 | AGOC 5’ TR InvPCR RV | 5’-TCTTGTTATAGATATCAGTTTAAACCCTAGG-3’ | - | 31 bp | 58.9°C |
| I1 | I1-1 | AGOC #1 ConPCR FD | 5’-TACAACAGAAAAGGGGAACTATATTGG-3’ | - | 27 bp | 60.6°C |
|  | I1-2 | AGOC #1 ConPCR RV | 5’-ATTAATAAATAAACCTCGATATACAGACCG-3’ | Identical with I2-2, I3-2, I4-2, I8-2 and I9-2 | 30 bp | 59.3°C |
| I2 | I2-1 | AGOC #3 ConPCR FD | 5’-AAAAGTTACAGACCCTGTACACAAATG-3’ | - | 27 bp | 59.6°C |
|  | I2-2 | AGOC #3 ConPCR RV | 5’-ATTAATAAATAAACCTCGATATACAGACCG-3’ | Identical with I1-2, I3-2, I4-2, I8-2 and I9-2 | 30 bp | 59.3°C |
| I3 | I3-1 | AGOC #5 ConPCR FD | 5’-TAAATCGGTTTCTTTGTCGCG -3’ | - | 21 bp | 60.9°C |
|  | I3-2 | AGOC #5 ConPCR RV | 5’-ATTAATAAATAAACCTCGATATACAGACCG-3’ | Identical with I1-2, I2-2, I4-2, I8-2 and I9-2 | 30 bp | 59.3°C |
| I4 | I4-1 | AGOC #6 ConPCR FD | 5’-GACACCCTGTACAACAATTCCAAC-3’ | - | 24 bp | 60.4°C |
|  | I4-2 | AGOC #6 ConPCR RV | 5’-ATTAATAAATAAACCTCGATATACAGACCG-3’ | Identical with I1-2, I2-2, I3-2, I8-2 and I9-2 | 30 bp | 59.3°C |
| I5 | I5-1 | AGOC{ATub'#O(LA)-mEmerald} #1 ConPCR FD | 5'-GCATGATTATCTTTAACGTACGTCAC-3' | Identical with I6-1 and I7-1 | 26 bp | 58.9°C |
|  | I5-1 | AGOC{ATub'#O(LA)-mEmerald} #1 ConPCR RV | 5'-AATCGAGAATGGTCCTTATCAGTG-3' | - | 24 bp | 59.4°C |
| I6 | I6-1 | AGOC{Zen1'#O(LA)-mEmerald} #1 ConPCR FD | 5'-GCATGATTATCTTTAACGTACGTCAC-3' | Identical with I5-1 and I7-1 | 26 bp | 58.9°C |
|  | I6-2 | AGOC{Zen1'#O(LA)-mEmerald} #1 ConPCR RV | 5'-TGACCAGGACTATGCCTACCTTATAC-3' | - | 26 bp | 59.7°C |
| I7 | I7-1 | AGOC{Zen1'#O(LA)-mEmerald} #2 ConPCR FD | 5'-GCATGATTATCTTTAACGTACGTCAC-3' | Identical with I5-1 and I6-1 | 26 bp | 58.9°C |
|  | I7-2 | AGOC{Zen1'#O(LA)-mEmerald} #2 ConPCR RV | 5'-AAACAGCCGGCTAAAAATCC-3' | - | 20 bp | 59.1°C |
| I8 | I8-1 | AGOC{ATub'SiaTr-mEmerald} #1 ConPCR FD | 5'-AATTATAAAATAAGCTGAACCTTTGATGTAG-3' | - | 31 bp | 59.0°C |
|  | I8-2 | AGOC{ATub'SiaTr-mEmerald} #1 ConPCR FD | 5'-ATTAATAAATAAACCTCGATATACAGACCG-3' | Identical with I1-2, I2-2, I3-2, I4-2 and I9-2 | 30 bp | 59.3°C |
| I9 | I9-1 | AGOC{ATub'H2B-mEmerald} #1 ConPCR FD | 5'-AGTTGCAAAATAGGGTTAGAAACCTAG-3' | - | 27 bp | 59.4°C |
|  | I9-2 | AGOC{ATub'H2B-mEmerald} #1 ConPCR RV | 5'-ATTAATAAATAAACCTCGATATACAGACCG-3' | Identical with I1-2, I2-2, I3-2, I4-2 and I8-2 | 30 bp | 59.3°C |
