# Supplementary material for: A universal vector concept for a direct genotyping of transgenic organisms and a systematic creation of homozygous lines
Source: eLife. 2018 Mar 15;7:e31677. doi: 10.7554/eLife.31677 (PMC5854464; doi:10.7554/eLife.31677)
Supplement: Supplementary file 10. — In total, 7 transgenic lines with 21 sublines were created, that is, 6 proof-of-principle AGOC sublines, 13 functional AGOC sublines and 2 helper sublines. Two of the functional AGOC sublines have been analyzed with live imaging previously, live imaging data for three more is provided in this study. [file elife-31677-supp10.docx]

| **Vector** | **Subline** | **Insertion location known** | **AGOC procedure** | **Fluorescence signal** | **Comment** |
| --- | --- | --- | --- | --- | --- |
| pAGOC | AGOC #1 | Yes (Supplementary file 3) | Successful | mO/mC – strong | - |
|  | AGOC #2 | No | Successful | mO/mC – strong | - |
|  | AGOC #3 | Yes (Supplementary file 3) | Successful | mO/mC – strong | - |
|  | AGOC #4 | No | Successful | mO/mC – strong | - |
|  | AGOC #5 | Yes (Supplementary file 3) | Successful | mO/mC – moderate | - |
|  | AGOC #6 | Yes (Supplementary file 3) | Successful | mO/mC – strong (slight enhancer trap pattern) | - |
| pAGOC{ATub’#O(LA)-mEmerald} | AGOC{ATub’#O(LA)-mEmerald} #1 | Yes (Supplementary file 3) | Successful | mO/mC – strong, mEmerald – strong | - |
| pAGOC{Zen1’#O(LA)-mEmerald} | AGOC{Zen1’#O(LA)-mEmerald} #1 | Yes (Supplementary file 3) | Successful | mO/mC – strong, mEmerald – moderate | - |
|  | AGOC{Zen1’#O(LA)-mEmerald} #2 | Yes (Supplementary file 3) | Successful | mO/mC – strong, mEmerald – strong | Live imaging in this study |
|  | AGOC{Zen1’#O(LA)-mEmerald} #3 | No | Successful | mO/mC – moderate, mEmerald – weak | Initially a nested insert |
| pAGOC{ARP5’#O(LA)-mEmerald} | AGOC{ARP5’#O(LA)-mEmerald} #1 | No | Successful | mO/mC – strong, mEmerald – strong | Live imaging in this study |
|  | AGOC{ARP5’#O(LA)-mEmerald} #2 | No | Successful | mO/mC – strong, mEmerald – strong | Live imaging in this study |
| pAGOC{ATub’SiaTr-mEmerald} | AGOC{ATub’SiaTr-mEmerald} #1 | Yes (Supplementary file 3) | Successful | mO/mC – strong, mEmerald – not detectable | - |
|  | AGOC{ATub’SiaTr-mEmerald} #2 | No | Successful | mO/mC – strong, mEmerald – not detectable | - |
|  | AGOC{ATub’SiaTr-mEmerald} #3 | No | Successful | mO/mC – strong, mEmerald – not detectable | - |
| pAGOC{ATub’H2B-mEmerald} | AGOC{ATub’H2B-mEmerald} #1 | Yes (Supplementary file 3) | Successful | mO/mC – strong, mEmerald – moderate (enhancer trap pattern) | Live imaging data published in Strobl et al., 2017b |
|  | AGOC{ATub’H2B-mEmerald} #2 | No | Successful | mO/mC – weak, mEmerald – weak | - |
|  | AGOC{ATub’H2B-mEmerald} #3 | No | Successful | mO/mC – strong, mEmerald – moderate | - |
|  | AGOC{ATub’H2B-mEmerald} #4 | No | Unsuccessful | mO/mC – strong, mEmerald – strong | Live imaging data published in Strobl et al., 2017b |
| pICE[HSP68’NLS-Cre} | ICE[HSP68’NLS-Cre} #1 | No | - | mCe – strong | Subline is homozygous |
|  | ICE[HSP68’NLS-Cre} #2 | No | - | mCe – strong | Subline is homozygous |
