# Supplementary material for: A universal vector concept for a direct genotyping of transgenic organisms and a systematic creation of homozygous lines
Source: eLife. 2018 Mar 15;7:e31677. doi: 10.7554/eLife.31677 (PMC5854464; doi:10.7554/eLife.31677)
Supplement: Supplementary file 11. — All components were obtained from AHF Analysentechnik, Tübingen, Germany. [file elife-31677-supp11.docx]

| **Filter set** | **Excitation filter** | **Beam splitter** | **Emission filter** | **Comment** |
| --- | --- | --- | --- | --- |
| for mCerulean (mCe FS) | 436/20 ET Bandpass  (F49-436) | 455 nm  (F48-455) | 480/40m ET Bandpass  (F47-480) | - |
| for mOrange  (mO FS) | 546/10 ET Bandpass  (F49-547) | 565 nm  (F33-565V20) | 575/15 BrightLine HC  (F39-575) | Blocks mCherry fluorescence nearly completely |
| for mCherry  (mC FS) | 586/15 BrightLine HC  (F37-589) | 595 nm  (F43-595V20) | 645/75 ET Bandpass  (F47-645) | Blocks mOrange fluorescence nearly completely |
