# Supplementary material for: A universal vector concept for a direct genotyping of transgenic organisms and a systematic creation of homozygous lines
Source: eLife. 2018 Mar 15;7:e31677. doi: 10.7554/eLife.31677 (PMC5854464; doi:10.7554/eLife.31677)
Supplement: Supplementary file 12. [file elife-31677-supp12.docx]

| **Dataset (DS)** | **DS0001** | **DS0002** | **DS0003** |
| --- | --- | --- | --- |
| Species | *Tribolium castaneum* (Herbst)  Arthropoda → Insecta → Coleoptera → Tenebrionidae | | |
| Line | AGOC{Zen1’  #O(LA)-mEmerald}  #1 subline | AGOC{ARP5’  #O(LA)-mEmerald}  #1 subline | AGOC{ARP5’  #O(LA)-mEmerald}  #2 subline |
| Line Genotype | one insert  (mC/mC) homozygous | one insert  (mC/mC) homozygous | one insert  (mC/mC) homozygous |
| Stock | ~500 adults, less than 1 months old | | |
| Stock Medium | full grain wheat flour (113061006, Demeter, Darmstadt, Germany)  supplemented with 5% (wt/wt) inactive dry yeast  (62-106, Flystuff, San Diego, CA, USA) | | |
| Stock Conditions | 12:00 h light / 12:00 h darkness at 25°C and 70% relative humidity  (DR-36VL, Percival Scientific, Perry, IA, USA) | | |
| Egg Laying Period | 01:00 h at 25°C and 70% relative  humidity exposed to light | | |
| Egg Laying Medium | 405 fine wheat flour (113061036, Demeter, Darmstadt, Germany)  supplemented with 5% (wt/wt) inactive dry yeast  (62-106, Flystuff, San Diego, CA, USA) | | |
| Pre-imaging Incubation | 15:00 h at 25°C and 70% relative humidity in darkness, 01:00 h at room temperature (23±1°C) | | |
| LSFM Type | DSLM (digital scanned laser light sheet-based fluorescence microscope – Keller et al., 2008) | | |
| Laser Lines | 488 nm / 20 mW diode laser  (PhoxX 488-20, Omicron Laserprodukte GmbH, Rodgau-Dudenhofen, Germany) | | |
| Excitation Objective | 2.5× NA 0.06 EC Epiplan-Neofluar objective (422320-9900-000**,** Carl Zeiss, Göttingen, Germany) | | |
| Emission Objective | 10× NA 0.3 W N-Achroplan objective (420947-9900-000, Carl Zeiss, Göttingen, Germany) | | |
| Emission Filters | 525/50 single-band bandpass filter (FF03-525/50-25, Semrock/AHF Analysentechnik AG, Tübingen, Germany) | | |
| Camera | High-resolution CCD (Clara, Andor, Belfast, United Kingdom), 14 bit, 1040×1392 pixel (pitch 6.45 µm) | | |
| Dataset File Type | TIFF, 16 bit grayscale (planes saved as Z stacks in ZIP-compressed container files, indicated as PL(ZS)) | | |
| Dechorionation | ~60-90 s in 10% (vol/vol) sodium hypochlorite  (425044-250ML, Sigma Adlrich, Taufkirchen, Germany)  in PBS pH 7.4 (10010-023, Gibco Life Technologies GmbH, Darmstadt, Germany) | | |
| Mounting Method | Cobweb holder (embryos are glued to a thin agarose film spanning a slotted hole – Strobl et al., 2017b) | | |
| Mounting Agarose | 1% (wt/vol) low-melt agarose (6351.2, Carl Roth, Karlsruhe, Germany)  in PBS pH 7.4 (10010-023, Gibco Life Technologies GmbH, Darmstadt, Germany) | | |
| Imaging Buffer | PBS pH 7.4 (10010-023, Gibco Life Technologies GmbH, Darmstadt, Germany) | | |
| Imaging Temperature | room temperature (23±1°C) | room temperature (23±1°C) | room temperature (23±1°C) |
| Retrieval | developed to healthy adult | developed to healthy adult | developed to healthy adult |

| **Dataset (DS)** | **DS0001** | **DS0002** | **DS0003** |
| --- | --- | --- | --- |
| Dataset Size | 14.1 Gigabyte (TIFF) | 57.5 Gigabyte (TIFF) | 72.5 Gigabyte (TIFF) |
| Figures | 5A | 5B, C, D | 5D |
| Supplementary Videos | 1 | 2 | 3 |
| Comment | - | - | - |
| **Time Points (TP)** | **49 (TP0001-TP0049)** | **193 (TP0001-TP0193)** | **241 (TP0001-TP0241)** |
| TP Interval | 00:30 h | 00:30 h | 00:30 h |
| Total Time (TP×TP Interval) | 24:00 h | 96:00 h | 120:00 h |
| **Directions (DR)** | **4 (DR0001-DR0004)** | **4 (DR0001-DR0004)** | **4 (DR0001-DR0004)** |
| DR Orientations | 0°, 90°, 180°, 270° | 0°, 90°, 180°, 270° | 0°, 90°, 180°, 270° |
| **Channels (CH)** | **1 (CH0001)** | **2 (CH0001-CH0002)** | **1 (CH0001)** |
| CH0001 Excitation | 488 nm | 488 nm | 488 nm |
| CH0001 Power | 135 µW (close to the embryo) | 270 µW (close to the embryo) | 270 µW (close to the embryo) |
| CH0001 Exposure Time | 50 ms | 50 ms | 50 ms |
| CH0001 Emission Filter | 525/50 single-band bandpass filter | 525/50 single-band bandpass filter | 525/50 single-band bandpass filter |
| **Planes (PL)** | **150 (PL0001-PL0150)** | **150 (PL0001-PL0150)** | **150 (PL0001-PL0100)** |
| Z Spacing | 2.58 µm | 2.58 µm | 2.58 µm |
| Z Distance (PL×Z Spacing) | 387.0 µm | 387.0 µm | 387.0 µm |
| **X-Dimensions (XD)** | **600 pixels (cropped)** | **600 pixels (cropped)** | **600 pixels (cropped)** |
| X Spacing | 0.645 µm | 0.645 µm | 0.645 µm |
| X Length (XD×X Spacing) | 387.0 µm | 387.0 µm | 387.0 µm |
| **Y-Dimensions (YD)** | **1000 pixels (cropped)** | **1000 pixels (cropped)** | **1000 pixels (cropped)** |
| Y Spacing | 0.645 µm | 0.645 µm | 0.645 µm |
| Y Length (YD×Y Spacing) | 645.0 µm | 645.0 µm | 645.0 µm |

All datasets can be downloaded at www.physikalischebiologie.de/bugcube.
