# Supplementary material for: A universal vector concept for a direct genotyping of transgenic organisms and a systematic creation of homozygous lines
Source: eLife. 2018 Mar 15;7:e31677. doi: 10.7554/eLife.31677 (PMC5854464; doi:10.7554/eLife.31677)
Supplement: Transparent reporting form [file elife-31677-transrepform.docx]

***eLife’s* transparent reporting form**

We encourage authors to provide detailed information *within their submission* to facilitate the interpretation and replication of experiments. Authors can upload supporting documentation to indicate the use of appropriate reporting guidelines for health-related research (see [EQUATOR Network](http://www.equator-network.org/%20)), life science research (see the [BioSharing Information Resource](https://biosharing.org/)), or the [ARRIVE guidelines](http://www.plosbiology.org/article/info:doi/10.1371/journal.pbio.1000412) for reporting work involving animal research. Where applicable, authors should refer to any relevant reporting standards documents in this form.

If you have any questions, please consult our Journal Policies and/or contact us: [editorial@elifesciences.org](mailto:editorial@elifesciences.org).

**Sample-size estimation**

- You should state whether an appropriate sample size was computed when the study was being designed
- You should state the statistical method of sample size computation and any required assumptions
- If no explicit power analysis was used, you should describe how you decided what sample (replicate) size (number) to use

Please outline where this information can be found within the submission (e.g., sections or figure legends), or explain why this information doesn’t apply to your submission:

Prior to the experiments, the sample size was estimated.

The crossing procedure relied on seven specific crossing steps with four experimental groups (see “Group Allocation”) that span 23 independent runs: **Table 1; Supplementary Tables 5, 6 and 7.**

For each crossing step, progeny number relied on the particular fertility of each adult pair, which usually produce between 50 and 150 descendants. In total, about 15,000 individuals were considered: **Table 1; Supplementary Tables 5, 6 and 7.**

**Replicates**

- You should report how often each experiment was performed
- You should include a definition of biological versus technical replication
- The data obtained should be provided and sufficient information should be provided to indicate the number of independent biological and/or technical replicates
- If you encountered any outliers, you should describe how these were handled
- Criteria for exclusion/inclusion of data should be clearly stated
- High-throughput sequence data should be uploaded before submission, with a private link for reviewers provided (these are available from both GEO and ArrayExpress)

Please outline where this information can be found within the submission (e.g., sections or figure legends), or explain why this information doesn’t apply to your submission:

The crossing procedure was run 23 independent times, subdivided in four groups (see “Group Allocation”) à 6*, 4*, 6 and 7 transgenic sublines: **Table 1; Supplementary Tables 5, 6 and 7.**

* the AGOC #5 and #6 sublines were each used in three crossing procedures with alternative conditions, equaling 10 independent runs

No data was excluded. We encountered one case of ‘incomplete recombination’ (**Table 1, second row**), which did not interfere with the procedure. A footnote is attached to **Table 1**. We encountered one case where one subline was not heterozygous fertile (**Supplementary Table 7, fourth row**). A respective footnote is attached to **Supplementary Table 7**.

**Statistical reporting**

- Statistical analysis methods should be described and justified
- Raw data should be presented in figures whenever informative to do so (typically when N per group is less than 10)
- For each experiment, you should identify the statistical tests used, exact values of N, definitions of center, methods of multiple test correction, and dispersion and precision measures (e.g., mean, median, SD, SEM, confidence intervals; and, for the major substantive results, a measure of effect size (e.g., Pearson's r, Cohen's d)
- Report exact p-values wherever possible alongside the summary statistics and 95% confidence intervals. These should be reported for all key questions and not only when the p-value is less than 0.05.

Please outline where this information can be found within the submission (e.g., sections or figure legends), or explain why this information doesn’t apply to your submission:

All raw data (*i.e.* scoring results) are provided in the manuscript: **Table 1; Supplementary Tables 5, 6 and 7.**

(For large datasets, or papers with a very large number of statistical tests, you may upload a single table file with tests, Ns, etc., with reference to sections in the manuscript.)

**Group allocation**

- Indicate how samples were allocated into experimental groups (in the case of clinical studies, please specify allocation to treatment method); if randomization was used, please also state if restricted randomization was applied
- Indicate if masking was used during group allocation, data collection and/or data analysis

Please outline where this information can be found within the submission (e.g., sections or figure legends), or explain why this information doesn’t apply to your submission:

Groups were defined according to the vectors that the sublines carry:

Group 1 (proof-of-principle): **Table 1**	(6 sublines*)

Group 2 (swapped genders and alt. Cre line): **Supplementary Table 5**	(4 sublines*)

Group 3 (carrying the default Lifeact tag): **Supplementary Table 6**	(6 sublines)

Group 4 (alternative endogenous label): **Supplementary Table 7**	(7 sublines)

* the AGOC #5 and #6 sublines were each used in three crossing procedures with alternative conditions

About 40% of the scores, at randomly chosen steps during the mating procedure, were determined by experienced, but otherwise uninitiated technical assistants.

**Additional data files (“source data”)**

- We encourage you to upload relevant additional data files, such as numerical data that are represented as a graph in a figure, or as a summary table
- Where provided, these should be in the most useful format, and they can be uploaded as “Source data” files linked to a main figure or table
- Include model definition files including the full list of parameters used
- Include code used for data analysis (e.g., R, MatLab)
- Avoid stating that data files are “available upon request”

Please indicate the figures or tables for which source data files have been provided:

All numerical data is provided within the **Table 1** and **Supplementary Tables 1, 2, and 5-7**. The raw data was collected in a comprehensive Excel sheet, which was uploaded: “**2017StroblStelzerAGOC-Rev1-Table1-SourceData1.xlsx**”. The formatting of the Excel sheet is different from the tables in the manuscript, the data is ordered by transgenic sublines, not by crossing steps.
